# Failed species, innominate forms, and the vain search for species limits: cryptic diversity in dusky salamanders (*Desmognathus*) of eastern Tennessee

**DOI:** 10.1002/ece3.780

**Published:** 2013-09-08

**Authors:** Stephen G Tilley, Joseph Bernardo, Laura A Katz, Lizmarie López, J Devon Roll, Renée L Eriksen, Justin Kratovil, Noëlle K J Bittner, Keith A Crandall

Ecology and Evolution 2013; 3(8): pages 2547–2567

doi: 10.1002/ece3.636

Table 2 reports incorrect GAPDH allozyme frequencies for the Lemon Gap form at Locality 61, which is actually fixed for GAPDH^4^, and for the Sinking Creek form at Locality 31, which is actually polymorphic for GAPDH^2^ and GAPDH^3^. We still assert that the allozyme frequencies at Locality 36 may reflect gene exchange with LGF.

A corrected version of Table 2 appears below. The text at the end of the Results section at the bottom of P. 15 should read as follows:

**Table 2:** Allozyme variation at diagnostic loci in the population at localities 73 and 36, the Lemon Gap form and the Sinking Creek form

	Clade γ Locality 73	Clade γ Locality 36	Lemon Gap form Locality 61	Sinking Creek form Locality 31
AK	*N* = 10	*N* = 7	*N* = 39	*N* = 5
2	0	1	1	0
3	1	0	0	1
GAPDH	*N* = 10	*N* = 7	*N* = 34	*N* = 5
1	1	0	0	0
2	0	0	0	0.800
3	0	1	0	0.200
4	0	0	1	0
GDH	*N* = 10	*N* = 6	*N* = 29	*N* = 5
1	0	1	0.759	0
2	1	0	0.241	1
LDH2	*N* = 9	*N* = 7	*N* = 45	*N* = 5
2	0	0.429	0.022	0
5	0	0.571	0.200	1
6	0	0	0.000	0
7	0.833	0	0.567	0
8	0.167	0	0.211	0
PEP	*N* = 10	*N* = 6	*N* = 45	*N* = 4
4	0	0.417	0.000	0
5	0.250	0	0.311	0
6	0	0	0.011	0
7	0	0.583	0.133	0
9	0.750	0	0.511	0.375
10	0	0	0.033	0
11	0.000	0	0.000	0.625
PGDH	*N* = 10	*N* = 7	*N* = 53	*N* = 5
1	0	0	0.009	0
2	0	0	0.009	0
3	0	1	0.840	1
4	1	0	0.142	0

“The populations at localities 36 and 73 are fixed or nearly fixed for the same variants at 16 presumptive loci, but exhibit complete differentiation at the remaining six (AK, GAPDH, GDH, LDH-2, PEP, and PGDH). At each of these diagnostic loci except GAPDH, the population at Locality 36 shares a variant with LGF at Locality 61 (Table 2).”

